# Are healthcare professionals delivering opportunistic behaviour change interventions? A multi-professional survey of engagement with public health policy

**DOI:** 10.1186/s13012-018-0814-x

**Published:** 2018-09-21

**Authors:** Chris Keyworth, Tracy Epton, Joanna Goldthorpe, Rachel Calam, Christopher J. Armitage

**Affiliations:** 1 Manchester Centre for Health Psychology, Division of Psychology and Mental Health, School of Health Sciences, Faculty of Biology, Medicine and Health, University of Manchester, Manchester Academic Health Science Centre, Coupland 1 Building–Room G3, Oxford Road, Manchester, M13 9PL UK; 2 Manchester Centre for Health Psychology, Division of Psychology and Mental Health, School of Health Sciences, Faculty of Biology, Medicine and Health, University of Manchester, Manchester Academic Health Science Centre, Coupland 1 Building, Oxford Road, Manchester, M13 9PL UK; 3 NIHR Manchester Biomedical Research Centre, Manchester University NHS Foundation Trust, Manchester Academic Health Science Centre, Manchester, UK; 4 NIHR Greater Manchester Patient Safety Translational Research Centre, Manchester, UK

**Keywords:** Health promotion, Healthcare professionals, Health policy, Professional practice

## Abstract

**Background:**

“Making Every Contact Count” (MECC), a public health policy in the UK, compels healthcare professionals to deliver opportunistic health behaviour change interventions to patients during routine medical consultations. Professionals’ awareness of, and engagement with, the policy is unclear. This study examined (1) awareness of the MECC policy, and (2) the prevalence of MECC-related practice in relation to (a) perceived patient benefit, (b) how often healthcare professionals deliver interventions during routine consultations, and (c) the time spent on this activity.

**Methods:**

Cross-sectional national survey was administered in 2017 of 1387 healthcare professionals working in the UK’s National Health Service (NHS). Descriptive statistics were used to assess awareness and practice consistent with the MECC policy. Chi-square was used to gauge the potential representativeness of our sample compared to NHS employment data.

**Results:**

31.4% of healthcare professionals reported having heard of the policy; nevertheless, healthcare professionals perceived a need to provide patients with opportunistic behaviour change interventions in 55.9% (32,946/58,906) of consultations. However, healthcare professionals did not deliver interventions on 50.0% of occasions in which they perceived a need. Where behaviour change interventions were delivered to patients, this constituted 35.3% of the appointment time.

**Conclusions:**

Policy makers must address the gap between the proportion of patients that healthcare professionals perceive would benefit from opportunistic behaviour change interventions and those receiving them (an estimated 50.0%; 16,473 additional patients could have benefited). Future research should consider how healthcare professionals identify patients who might benefit from opportunistic behaviour change interventions and developing training for efficient delivery of interventions.

## Highlights

- Of the total sample, 436 healthcare professionals (31.4%) reported having heard of the “Making Every Contact Count” policy, suggesting awareness of this important public health policy is low.
- Even when healthcare professionals perceive a patient benefit (for approximately 32,946 out of a total 58,906 patients), they do not “Make Every Contact Count” in 50.0% of cases.
- Policy makers must increase awareness of “Making Every Contact Count” and ensure that strategies are in place to support healthcare professional practice in this domain.

## Background

In 2014, the National Institute for Health and Care Excellence (NICE) estimated that the total cost of physical inactivity, smoking, alcohol misuse and overweight to the NHS to be approximately £12,601 million [1]. Healthcare professionals are ideally placed to support and facilitate behaviour change with patients because of their frequent one-to-one patient contact. Delivery of opportunistic behaviour change interventions by healthcare professionals is both effective and cost-effective [2] with the cost of delivery falling below the cost per quality-adjusted life-year (QALY) agreed thresholds [3].

Public health policies are used internationally to encourage healthcare professionals to deliver behaviour change interventions [4, 5]. However, organisations face a number of barriers to implementing public health policy in relation to clinical practice [6–9]. For example, research examining implementation of NICE guidelines identified key implementation barriers; these include a lack of clinician engagement and lack of clarity of the personal relevance of the policy [6], perceived lack of time and resources to implement policy [6, 7] and a lack of managerial support and complex guidelines leading to poor implementation [8, 9]. Such barriers are consistent with both traditional and more recent theoretical approaches to understanding implementation [10]. For example Normalization Process Theory [11] can be used to understand how policy becomes embedded in practice, outlining the importance of engaging with public health policies (cognitive participation) and understanding how healthcare professionals make sense of the policy in question (coherence). Similarly, Michie et al.’s COM-B model of behaviour [12] states that individuals must have (1) the capability (possessing the skills and engaging in the necessary thought processes), (2) the opportunity (including time, resources and support from colleagues), and (3) the motivation (having the desire) to engage in practice consistent with policy recommendations.

Making Every Contact Count (MECC), a National Health Service (NHS) policy aimed at patient-facing healthcare professionals, emphasises the prevention of health problems being at the heart of every NHS contact [13, 14]. MECC policy, based on recognised behaviour change evidence [12], is developed alongside a wide-ranging list of partner organisations including local authorities, Public Health England, the Royal Society for Public Health, the Care Quality Commission and NICE [13]. Attempts to enforce the MECC policy include laying out the principles as a key component of the NHS standard contract [15], which relates to the commissioning and delivery of healthcare services, and requires healthcare providers to develop and maintain an organisational plan for MECC-related practice. The policy compels healthcare professionals to use the millions of day-to-day clinical interactions with patients to offer concise health behaviour change interventions, encourage people to change their behaviour and to direct them to local services that can support them. As a minimum, all healthcare professionals in direct contact with patients are advised to “raise awareness, motivate and signpost people to help them improve their health and wellbeing” [13].

Research suggests that opportunistic behaviour change interventions delivered by healthcare professionals can result in patient behaviour change [16–18], but opportunities to address health behaviours directly during routine clinical interactions are often missed [19–23]. Precise reasons for failing to address behaviour change in the consultation may include a lack of training [24], healthcare professionals’ own beliefs about patient motivation to change their behaviour [25–27] or wider barriers such as time, workload or organisational barriers [28–30]. However previous research is limited by focusing on prescribed behaviour change interventions delivered by defined healthcare professionals managing specific health conditions (e.g. smoking cessation interventions delivered by dentists [31]) in the context of research studies rather than on opportunistic behaviour change interventions delivered in routine practice [32, 30].

The present study aims to assess for the first time: (1) whether healthcare professionals are aware of the MECC policy; (2) perceptions about how many of their patients would benefit from opportunistic behaviour change interventions; (3) of the patients who would benefit, with how many could healthcare professionals deliver opportunistic behaviour change interventions in routine consultations, and (4) how much appointment time is spent on delivering opportunistic behaviour change interventions in the context of patients who may benefit.

## Methods

### Design and procedure

A cross-sectional survey design was used. Healthcare professionals with a patient-facing role were recruited via a survey panel company (YouGov) in 2017. A purposive sample of healthcare professionals working in the National Health Service (NHS) in the United Kingdom was invited to take part in an online questionnaire and were incentivised in accordance with YouGov’s points system, whereby respondents accumulate points for taking part in online surveys, which can be exchanged for cash or entry into a prize draw [33]. The data were collated by YouGov and sent securely to the research team for analysis.

### Participants

A range of healthcare professionals were recruited and included general practitioners (GPs); specialist doctors; nurses; midwives, and scientific, therapeutic and technical staff (e.g. pharmacists, psychologists, speech and language therapists). The sampling frame aimed to obtain the widest possible variation in participants according to demographic characteristics. Due to the questionnaire being targeted at patient-facing staff, we sought to recruit additional GPs as part of the sampling frame. Ethical approval was obtained from a university ethics committee (ref 2017-0739-1780), and informed consent was obtained from participants at the beginning of the questionnaire.

### Measures

The questionnaire, as part of a wider survey, collected demographic information such as gender and age, healthcare setting (e.g. primary care, secondary care) as well as the number of patients seen by the healthcare professional in a typical week. Participants were asked about their awareness of the MECC policy (a description of which was provided after participants answered) and about the extent to which they engaged in this activity as part of their daily practice. Participants were asked to rate (using a 0–100% rating scale): (a) what proportion of patients they saw would benefit from opportunistic behaviour change interventions, (b) the proportion of times they delivered opportunistic behaviour change interventions to the patients they thought would benefit, and (c) how much of their contact time they spent delivering opportunistic behaviour change interventions to the patients they thought would benefit.

### Analyses

Descriptive statistics were used to quantify MECC-related activities, and mean ratings of awareness of the MECC policy and MECC-related practices (the proportion of patients that would benefit from behaviour change interventions, the proportion to whom they deliver interventions and the amount of time spent on this activity) were calculated. Results are presented both across and within healthcare professional groups. Chi-square was used to gauge the potential representativeness of our sample compared to NHS employment data.

## Results

### Sample characteristics

The total sample (*n* = 1387) included nurses and health visitors (*n* = 438); general practitioners (*n* = 332); specialist doctors (*n* = 125) and scientific, therapeutic, and technical staff (*n* = 270). Healthcare professionals mostly worked in primary care (*n* = 339), acute care (*n* = 576) or community care (*n* = 257). Table 1 shows an overview of our sample compared to NHS data. Our sample includes the intended over-representation of GPs and under-representation of support staff; other staff groups in our sample more closely resemble proportions of staff groupings in the NHS. In a typical week, healthcare professionals reported seeing a mean of 50 patients as part of routine consultations (range 40–70 across healthcare professional groups), spending on average 31 min with each patient (range 15–48 min across groups).

**Table 1:** Sample characteristics

Variable	*n*	(%)	Mean	(SD)	NHS data (%)^a,d^	X2 for difference between sample and population
Gender (%)						
Male	446	(32.2)			(23.0)	66.18 (*p* < .001)
Female	941	(67.8)			(77.0)	66.18 (*p* < .001)
Total	1387					
Age, years			45	(11.46)	43.0^f^	
Healthcare professional group^a^						
General practitioners	332	(23.9)			(3.9)	1471.06 (*p* < .001)
Specialist doctors	125	(9.0)			(12.9)	18.76 (*p* < .001)
Nurses and health visitors	438	(31.6)			(32.1)	0.16 (*p* = .69)
Midwives	42	(3.0)			(2.5)	1.42 (*p =* .23)
Ambulance staff	20	(1.4)			(2.0)	2.55 (*p =* .11)
Scientific, therapeutic and technical staff^b^	270	(19.5)			(14.0)	34.80 (*p* < .001)
Nurses working in GP practices	88	(6.3)			(1.9)	143.57 (*p* < .001)
Support to clinical staff	49	(3.5)			(29.7)	455.77 (*p* < .001)
Other HCHS staff/unknown classifications	23	(1.3)			(1.1)	0.51 (*p* = .48)
Total	1387					
Setting^e^				–		
NHS acute care	576	(41.5)				
NHS tertiary care	83	(6.0)				
NHS community care	257	(18.5)				
NHS primary care	339	(24.4)				
Other	132	(9.5)				
Total	1387					
How many service users do you see in a typical week?			50	(31.89)	–	
General practitioners	70	(25.34)				
Specialist doctors	53	(32.13)				
Nurses and health visitors	41	(29.22)				
Midwives	46	(26.43)				
Ambulance staff	40	(25.28)				
Scientific, therapeutic and technical staff^b^	40	(31.24)				
Nurses working in GP practices	60	(32.59)				
Support to clinical staff	45	(35.87)				
Other HCHS staff/unknown classifications	46	(41.40)				
Total number of service users seen by all included healthcare professionals	58,906^c^					
How many minutes do you spend on average with each service user? (minutes)					–	
General practitioners			22	(15.92)		
Specialist doctors			25	(14.13)		
Nurses and health visitors			37	(19.96)		
Midwives			37	(16.87)		
Ambulance staff			48	(17.15)		
Scientific, therapeutic and technical staff^b^			36	(20.15)		
Nurses working in GP practices			26	(18.03)		
Support to clinical staff			35	(20.15)		
Other HCHS staff/unknown classifications			15	(19.75)		
Total			31	(19.54)		

^a^Staff categories and NHS data according to NHS digital workforce statistics (headcount), excludes NHS infrastructure support and admin staff; https://digital.nhs.uk/data-and-information/publications/statistical/healthcare-workforce-statistics/healthcare-workforce-statistics-march-2017-experimental

^b^Includes pharmacists (*n* = 33), psychologists (*n* = 34), speech and language therapists (*n* = 23), radiographers (*n* = 33), physiotherapists (*n* = 26) and occupational therapists (*n* = 36)

^c^Participants were asked to estimate the number of patients they would see in a typical week; therefore, this is an approximate number only, based on *n* = 1177 healthcare professionals who provided an estimate

^d^Data regarding age and gender retrieved from NHS employers: http://www.nhsemployers.org/~/media/Employers/Publications/Gender%20in%20the%20NHS

^e^National data unavailable from NHS digital

^f^Mean

### Awareness of “Making Every Contact Count”

Results are presented in Table 2. Thirty-one percent of the sample (*n* = 436) reported having heard of the MECC framework. Awareness was low across all professional groups (Table 3) (range 14.3–43.9%), particularly amongst GPs (17.2% of GPs had heard of the policy). Midwives were the group reporting the highest awareness (43.9% of midwives had heard of the policy), followed by nurses working in GP practices (42.5% reported awareness of the policy).

**Table 2:** Awareness and prevalence of “Making Every Contact Count” (*n* = 1387)

Question	*n*	(%)	Mean (%)	(SD)
(Awareness)				
Before today, had you heard of the Making Every Contact Count consensus statement?				
Yes	436	(31.4)		
No	830	(59.8)		
Do not know	83	(6.0)		
Did not state	38	(2.7)		
Total	1387			
(Prevalence)				
Of the service users you see in a typical working week, what proportion do you think would benefit from you Making Every Contact Count?			55.93	(31.86)
Of the service users you see in a typical working week, who you think would benefit**,** with what proportion do you Make Every Contact Count?			50.00	(31.34)
Of the service users you see in a typical working week who you think would benefit, how much of their appointment time do you spend with them making every contact count?			35.30	(30.92)

**Table 3:** Awareness and prevalence of “Making Every Contact Count” (by healthcare professional group)

	Healthcare professional group								
Question	General practitioners	Specialist doctors	Nurses and health visitors	Midwives	Ambulance staff	Scientific, therapeutic and technical staff	Nurses working in GP practices	Support to clinical staff	Other HCHS staff/unknown classifications
(Awareness)									
Before today, had you heard of the Making Every Contact Count consensus statement? (Yes)	57/331 (17.2%)	38/121 (31.4%)	171/421 (40.6%)	18/41 (43.9%)	4/19 (21.1%)	93/263 (35.4%)	37/87 (42.5%)	14/47 (29.8%)	2/14 (14.3%)
(Prevalence)									
Of the service users you see in a typical working week, what proportion do you think would benefit from you Making Every Contact Count?	*M* = 44.15% (*SD* = 29.55)	*M* = 54.88% (*SD* = 29.23)	*M* = 61.42% (*SD* = 31.25)	*M* = 71.61% (*SD* = 29.47)	*M* = 55.30% (*SD* = 34.25)	*M* = 58.41% (*SD* = 33.24)	*M* = 68.07% (*SD* = 29.59)	*M* = 68.37% (*SD* = 30.87)	*M* = 81.75% (*SD* = 23.42)
Of the service users you see in a typical working week, who you think would benefit, with what proportion do you Make Every Contact Count?	*M* = 33.97% (*SD* = 22.49)	*M* = 47.84% (*SD* = 32.09)	*M* = 59.81% (*SD* = 31.09)	*M* = 71.40% (*SD* = 23.28)	*M* = 35.10% (*SD* = 19.84)	*M* = 54.40% (*SD* = 35.01)	*M* = 65.36% (*SD* = 26.75)	*M* = 63.22% (*SD* = 34.07)	*M* = 70.40% (*SD* = 27.41)
Of the service users you see in a typical working week who you think would benefit, how much of their appointment time do you spend with them making every contact count?	*M* = 20.09% (*SD* = 18.24)	*M* = 27.86% (*SD* = 27.41)	*M* = 44.83% (*SD* = 31.91)	*M* = 48.04% (*SD* = 33.87)	*M* = 38.44% (*SD* = 25.65)	*M* = 36.98% (*SD* = 34.46)	*M* = 53.90% (*SD* = 32.36)	*M* = 58.90% (*SD* = 34.21)	*M* = 35.60% (*SD* = 25.15)

### Prevalence of “Making Every Contact Count”

Results are presented in Table 2. Healthcare professionals reported that 55.9% of patients whom they saw in a typical week would benefit from opportunistic behaviour change interventions (32,946 out of a total of 58,906 patients). Despite this, healthcare professionals felt able to deliver behaviour change interventions in just 50.0% (16,473/32,946) of such consultations. When behaviour change was discussed with patients, it took on average 35% of the consultation time.

Across professional groups (Table 3), the proportion of patients who would benefit from behaviour change interventions, according to healthcare professionals, ranged from 44.2–81.8%. The group reporting the lowest number of patients with whom behaviour change interventions would be beneficial were GPs (GPs reported that 44.2% of their patients would benefit). Amongst the highest group, midwives reported that 71.61% of their patients would benefit from a behaviour change intervention.

The group reporting the highest proportion of patients with whom they deliver behaviour change interventions was midwives (who reported delivering interventions to 71.4% of patients who would benefit). The lowest proportion (34% of patients) was reported by GPs. The amount of time dedicated to behaviour change during routine consultations ranged from 20.1 (reported by GPs) to 58.9% (reported by those working in roles providing support to clinical staff.

## Discussion

This is the first study to examine the extent to which healthcare professionals working in direct contact with patients reported delivering opportunistic behaviour change interventions consistent with a national public health policy [3, 13, 14]. There were two important findings. First, awareness of the MECC policy for delivering brief behaviour change interventions [13] was low; approximately one third of our sample reported having heard of the policy. Second, even when healthcare professionals perceived that patients would benefit from an opportunistic behaviour change intervention (approximately 32,946 out of a total 58,906 patients), they felt unable to “Make Every Contact Count” in 50.0% of these cases. Where behaviour change was discussed, this equated to 35% of the consultation time, suggesting a significant amount of time being invested in delivering behaviour change interventions, once a problem has been identified and there is an opportunity.

### Missed opportunities

Our data suggest that even though there was low awareness of the MECC policy, opportunistic behaviour change interventions are still delivered to patients during consultations. However, the question arises as to how healthcare professionals can identify the patients who would benefit from opportunistic behaviour change interventions and what is the content of the opportunistic behaviour change interventions that is being delivered.

Low awareness of policy, combined with low engagement with practice addressing behaviour change, is a cause for concern for both policy makers and intervention developers. Specific reasons contributing to the likelihood of behaviour change being part of the medical consultation may be due to a lack of appropriate training to deliver behaviour change interventions [24], fear of offending the patient [25] and health professionals’ beliefs about patient motivation [26, 27]. Specific reasons about why so many healthcare professionals were unaware of the MECC policy and the barriers to delivering opportunistic interventions should be the focus of future research.

Findings from the present study suggest that interventions should aim to support healthcare professionals with a patient-facing role in three key areas. First, to help identify patients who would benefit from opportunistic behaviour change advice (19% of our sample stated they did not know the proportion of their patients they thought would benefit from behaviour change). Second, to deliver opportunistic behaviour change interventions in a timely manner (given the proportion of the consultation time used to deliver behaviour change interventions, this may constitute a significant barrier). Third, to recognise where and when opportunistic behaviour change interventions have been successful (23% of our sample stated they did not know whether they had actually delivered a behaviour change intervention).

### Differences between professional groups

Public health policy [13] compels all patient-facing healthcare professionals to deliver opportunistic behaviour change interventions, yet awareness of the policy was low across all healthcare professional groups, and practice consistent with policy was low across most healthcare professional groups. The exception to this was amongst midwives. Whilst reporting low awareness of policy (43.9% of midwives reported awareness), they were the group reporting the highest proportion of patients with whom they deliver behaviour change interventions (71.4% of patients). It is widely recognised that pregnancy offers a “teachable moment” to address health behaviour change [34], yet our findings suggest there are still missed opportunities to deliver interventions to those in need (28.6% of patients could have received an intervention from a midwife). Additional concerns are the findings of low awareness of policy and low practice amongst healthcare professionals ideally placed to support behaviour change with patients, due to their frequent one-to-one contact with patients. This was particularly true amongst GPs; 17.2% of GPs reported awareness of the policy, reported the lowest proportion of patients with whom they deliver behaviour change interventions (34% of patients who would benefit) and reported spending the least amount of time during the consultation on this activity. A similar pattern was observed for nurses and health visitors, who reported low awareness of policy (40.6% of nurses reported being aware of the policy) and low practice (delivering behaviour change interventions to 59.8% of patients who would benefit).

### Implications for practice

Brief interventions delivered by healthcare professionals can result in small but significant changes to patients’ behaviour [16–18]. Healthcare professionals can deliver such brief interventions during routine consultations as part of routine medical practice at a relatively low cost [2]. Patient-facing healthcare professionals are particularly important as they enable interventions to have maximum reach, and evidence suggests these can be used effectively by healthcare professionals and incorporated into a time-restricted medical consultation [16, 17]. It may not seem feasible for healthcare professionals to deliver opportunistic behaviour change interventions, given the reported amount of time spent on this activity in the present study. However, behaviour change interventions can be delivered in as few as 30 s [17]. Future implementation strategies could draw upon recognised behaviour change theory, by enhancing healthcare professionals’ capabilities, opportunities and motivations to deliver interventions (as postulated by the COM-B model) in order to understand the influences on routine clinical behaviour. Specific behaviour change techniques can be used to support changes in clinician behaviour and increase implementation of public health policies by translating research evidence into practice [35–37]. The Behaviour Change Wheel (BCW) [38], proposed by Michie et al., comprises nine intervention functions that can be used to facilitate healthcare professional behaviour change (of which “persuasion” could be targeted to increase healthcare professionals engagement with delivering behaviour change interventions), and seven types of policy that can be used to deliver the intervention functions (of which “guidelines” and improvements in “service provision” could be targeted) [38].

Future research should aim to examine patient perceptions of MECC, and particularly, whether discussing behaviour change should be part of routine consultations. Research with GPs suggests that patients are not offended by the topic of weight management for example, with discussions being perceived as helpful [17]. Further, the advantages of interprofessional collaboration should be examined in future research, given the diverse groups of healthcare professionals that often work together to provide patient care. For example, increasing communication between healthcare professionals is recognised as an important aspect of delivery of effective patient care by the World Health Organization [39] and effective interprofessional collaboration may lead to improved communication between healthcare professionals, encourage joint decision making about patient care and lead to improvements in clinical practice [40, 41].

### Strengths and limitations

To our knowledge, this is the first attempt to examine the awareness and prevalence of “Making Every Contact Count”-related activities across healthcare professional groups using a large national sample. Findings highlight important opportunities to support healthcare professionals in identifying patients’ need for behaviour change interventions and to deliver time and cost effective interventions as part of routine medical consultations. Future research should aim to build on these findings and include measures of patient behaviour in order to examine the impact of such interventions on patient outcomes.

There are limitations to this study. Whilst we aimed to recruit a higher proportion of GPs, we had a lower proportion of clinical support staff, compared to national ratios relating to NHS roles (*X*^2^ values are presented in Table 1), which could be explained by the survey being primarily targeted at patient-facing staff. Additionally, in the absence of NHS data, we were unable to compare the precise timings each healthcare professional spends with their patient and the number of patients seen per week with that of national data. Whilst a recent survey suggests that GPs in the UK see an average of 41 patients per day [42] (compared to a reported 70 patients per week in our study) and a recent review suggests GP consultation rates are an average of 9.22 min [43] (compared to a reported 22 min in our study), research with nurses shows that contact time may vary considerably between 12 min to 1 h and 5 min [44]. Participants were identified from a pre-existing sample of healthcare professionals recruited and incentivised by YouGov to complete the questionnaire. The sample therefore may not be fully representative of the healthcare professionals working in the NHS as a whole. YouGov, however, attempted to overcome this by seeking the widest possible variation in participants according to demographic characteristics. Second, this study focused on awareness of policy and prevalence of policy-related practices only. Other factors that may not have been captured in the present study may be contributing to whether brief behaviour change interventions are delivered to patients. For example, there may be different priorities across the various healthcare specialisms, particularly those professionals working in secondary or specialist care roles, who may have more focused consultations and may perceive a lack of time to deliver opportunistic behaviour change interventions. Whilst this is a limitation of cross-sectional survey designs, we are now undertaking qualitative work that aims to capture the most relevant barriers to healthcare practice, beyond those commonly reported in the literature, such as time, workload and organisation barriers [28–30].

## Conclusions

Behaviour change is an issue of worldwide importance, and behaviour change interventions delivered opportunistically by healthcare professionals during routine practice have the potential to reduce morbidity and mortality. Healthcare professionals recognise the value and need for behaviour change interventions, but our findings suggest a lack of awareness of recommended practice guidelines in relation to this area of clinical practice. There may be important opportunities missed during routine practice for healthcare professionals to offer brief, opportunistic advice about behaviour change. By addressing these missed opportunities, this will take an important step towards prevention and management of long-term conditions in day-to-day clinical practice.

## Abbreviations

GP: General practitioner
MECC: Making Every Contact Count
NHS: National Health Service
NICE: National Institute for Health and Care Excellence
QALY: Quality-adjusted life-year
